# The efficacy, safety, and cost benefits of splints for fractures of the distal radius in children: A systematic review and meta-analysis protocol

**DOI:** 10.1097/MD.0000000000016562

**Published:** 2019-08-02

**Authors:** Xin Cui, Long Liang, Xu Wei, Xing Liao, Yongyao Li, Hao Cheng, Yanming Xie, Yongzhong Cheng, Yachao Du, Guangwei Liu, Hongyan Zhang, Shiheng Wang, Jiani Liu, Zhibo Wang, Yue Zhang, Yaliang Tian

**Affiliations:** a Wangjing Hospital; b Institute of Basic Research in Clinical Medicine, China Academy of Chinese Medical Sciences, Beijing; c Guangzhou University of Traditional Chinese Medicine, Guangzhou; d China Institute for History of Medicine and Medical Literature; e Xiyuan Hospital, China Academy of Chinese Medical Sciences, Beijing; f Longhua Hospital, Shanghai University of Traditional, Chinese Medicine, Shanghai, China.

**Keywords:** children, distal radius fractures, protocol, splints, systematic review

## Abstract

**Background:**

Distal radius fractures (DRFs) is one of the most common bone injuries in children, which may lead to deformity and other complications if the treatment is not prompt or appropriate. Splints external fixation is a common conservative treatment for such fractures. Therefore, we conducted a systematic review and meta-analysis to explore the efficacy, safety and cost benefits of splints in the treatment of DRFs in children.

**Methods:**

PubMed, Web of Science, Embase, Cochrane Library, Cochrane Central Register of Controlled Trials (CENTRAL), and ClinicalTrials.gov, Chinese National Knowledge Infrastructure Database (CNKI), Wanfang Database, and VIP Database were searched for eligible randomized controlled trials (RCTs). The methodological quality of the included studies and the level of evidence for results were assessed, respectively, using the risk bias assessment tool of Cochrane and the Grading of Recommendations Assessment, Development, and Evaluation (GRADE) method. Statistical analysis was conducted with Revman 5.3.

**Results:**

This study will analyze and integrate the existing evidence for effectiveness, safety and cost benefits of splints on DRFs in children.

**Conclusion:**

The conclusion of this study will provide evidence to effectiveness, safety and cost benefits of splints on DRFs in children, which can further guide the selection of appropriate interventions.

**PROSPERO registration number:**

CRD42019123429

## 1 Introduction

Distal radius fractures (DRFs) is one of the most common bone injuries in children, accounting for about 20% to 35% of all fractures in children.^[1–8]^ The incidence of DRFs in children in New Zealand is approximately 10.4 cases per 1000 children (3–15 years old) per year, which is equivalent to an average of 20 cases per day in a country with a population of 4 million.^[9]^

The 80% of DRFs in children are metaphyseal fractures, 50% of which affect only the radius, while the other 50% of cases affect both ulna and radius.^[3,10]^ The other 20% of children's distal radius fractures are characterized by epiphyseal fractures.^[3,10]^ Since the bone of children's wrist joint is cartilage, the nature of the bone is soft and the toughness is strong, so wrist joint injury is not common in children. But as wrist bones ossify, they become more prone to fractures and ligament damage. For adolescence, wrist joints that are almost completely ossified show a pattern of injury similar to those of adults.^[11,12]^

Freefall from 1 level to another, such as falling from playground equipment, is a major cause of upper limb fractures.^[13]^ Epidemiological studies have shown that both fall height and ground type have significant impacts on the risk of injury caused by falling of playground equipment.^[14,15]^ A major obstacle to reducing or avoiding injury to children is the lack of knowledge and information to effectively prevent injury.^[16]^

DRFs in children are usually treated in the emergency department of the hospital.^[17,18]^ Closed reduction with plaster splint or splints is the most common method for the treatment of such fractures,^[7]^ compared with adults, which is also relatively effective in children. However, the lack of relevant guidelines and high-level evidence-based research has affected doctors’ decision-making in clinical. Hence, it is necessary to conduct a systematic review of treatment for distal radius fractures in children with the increasing of related studies in recent years. In this study, integrated multiple existing randomized controlled trials (RCTs) to evaluate the clinical efficacy, safety, and cost benefits of splints in the treatment of DRFs in children, which may provide reference for clinical application.

## 2 Methods

### 2.1 Study registration

This protocol has been registered in the international prospective register of systematic reviews (PROSPERO), and the registration number is CRD42019123429. Available online: http://www.crd.york.ac.uk/PROSPERO/display_record.php?ID=CRD42019123429. The steps of this protocol will follow the Preferred Reporting Items for Systematic Review and Meta-analysis Protocols (PRISMA-P) statement guidelines.^[19]^ Since this study is a secondary literature study based on RCTs, no ethical approval is required.

### 2.2 Inclusion criteria for study selection

#### 2.2.1 Type of studies

We will only include RCTs. Retrospective studies, review, case reports, cohort studies, experimental studies, expert experience, and other non-RCTs will be excluded. There are no restrictions on languages.

#### 2.2.2 Type of participants

We will include studies that the children must be definitely diagnosed as distal radius fractures, not limited by gender, ethnicity, nationality, primary disease, or clinical stage, which was based on imaging diagnostic criteria.

#### 2.2.3 Type of interventions

We will include the studies that splint as an intervention in the experimental group, while we have no restrictions on intervention in the control group.

#### 2.2.4 Type of outcome measurements

##### 2.2.4.1 Primary outcomes

Visual analog scale (VAS) will be defined as the primary outcome to assess the degree of pain after fracture.

##### 2.2.4.2 Secondary outcomes

1. Grip strength, measured by any instrument.
2. Complications, measured by any instrument.
3. Patient and parent satisfaction and preference, measured by any instrument.
4. Cost-effectiveness analysis, measured by any instrument.
5. Behavioral activity ability, measured by the Activities Scale for Kids (ASK) questionnaire.
6. The degree of disability, measured by pediatric disability score (PDS).

### 2.3 Search strategy

Relevant literature was retrieved using multiple online databases including the PubMed, Web of Science, Embase, the Cochrane Library, the Cochrane Central Register of Controlled Trials (CENTRAL), ClinicalTrials.gov, the Chinese National Knowledge Infrastructure Database (CNKI), Wanfang and VIP Database. No limits were imposed on the dates, types, and statuses of the publications eligible for inclusion. The key terms used in the searches were: “radius fractures”, “Colles’ fracture”, “Smith’ fracture”, “Barton fracture”, “splint”, “static splints”, “static orthose”, “dynamic splints”, “dynamic orthoses”, “plintlet”, “wood splint”, “splintlet”, “splintage”, “small plywood”, “splint fixation”. Different search strategies were used for the Chinese and foreign language databases. In addition, the reference lists of previously published systematic reviews on the subject of splint for distal radius fractures in children were manually examined for further pertinent studies.

### 2.4 Selection of studies

Two reviewers independently read the title and abstract of the literature and screened the documents according to inclusion and exclusion criteria. When they are uncertain to determine whether to exclude, we will read the full text to identify the studies that need to be included.

### 2.5 Data extraction

**Figure 1:**
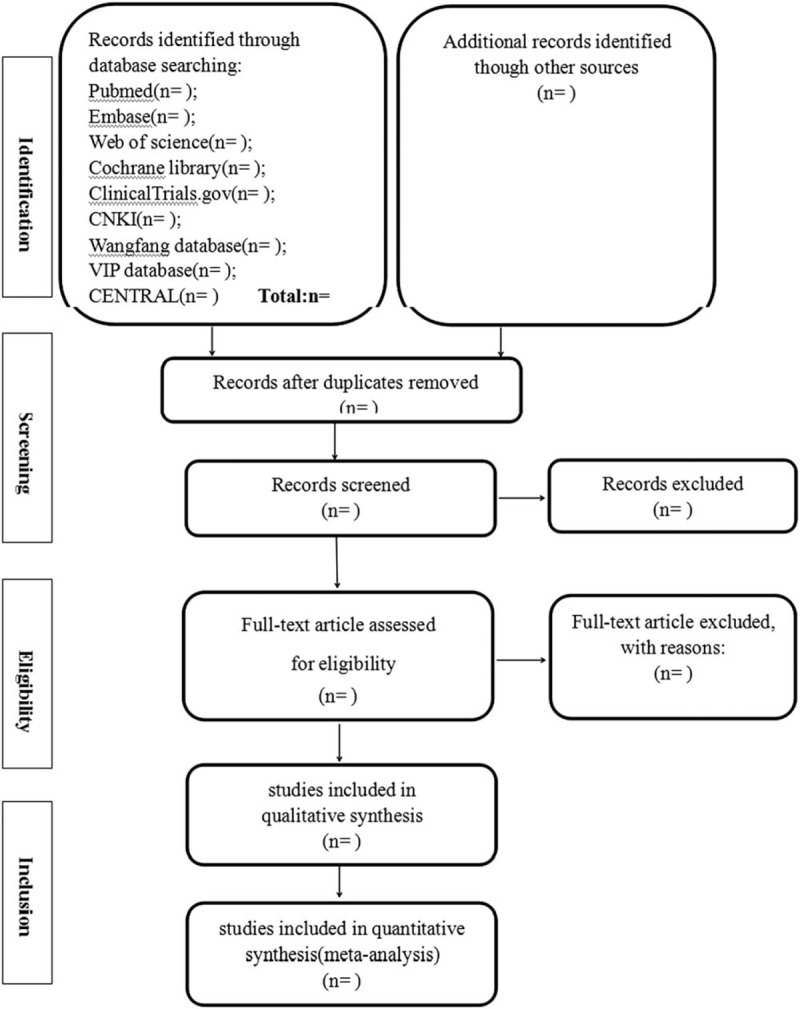
Flow diagram of study selection and screening process.

The following data will be independently extracted by 2 authors: the name of first author, year of publication, country, number of patients under total disc replacement, and lumbar fusion, sample size, age, gender of patients, disease course, follow-up duration. When relevant data has not been reported, we will contact the authors by email or in other ways to attempt to obtain the missing information. The review authors will resolve any disagreements by discussion, including input from a third independent review author if required. The Preferred Reporting Items for Systematic Review and Meta-analysis (PRISMA)'s flow diagram (Fig. 1) will be used to show the details of the study selection process.

### 2.6 Assessment of risk of bias

Two authors will assess the methodological quality of the included studies using the criteria outlined in the Cochrane Handbook for Systematic Reviews of Interventions 5.1.0.^[20]^ Two authors will also compare the results and will discuss any differences until agreement is reached. The domains to be assessed will include: random sequence generation, allocation concealment, blinding of participants and personnel, blinding of outcome assessment, incomplete outcome data, selective reporting, and other bias.

For other sources of bias, 2 aspects have been identified:

(1) trials stopped early owing to some data-dependent processes;
(2) baselines extreme imbalanced.

### 2.7 Measures of treatment effects

The outcomes of interest will include dichotomous data and continuous variables, Dichotomous data will be expressed as the risk ratio (RR), and mean difference (MD) will be used to assess differences in the continuous outcomes between the groups. Also, standardized mean difference (SMD) will be chosen if the clinical outcomes are the same, but have been measured using different methods in different trials. The corresponding 95% confidence interval (CI) for each parameter will be computed for the splint-treated group versus the control group. If quantitative synthesis is not appropriate, descriptive review will be selected.

### 2.8 Assessment of heterogeneity

Statistical heterogeneity across the included studies will be examined using the I^2^ statistic, with an I^2^ >50% regarded as being indicative of the possibility of statistical heterogeneity, resulting in the selection of a random-effects model for the computation of MD or SMD with its corresponding 95% CI. Otherwise, no obvious heterogeneity will be considered to be present in the included studies for values of I^2^ <50%, in which case the fixed-effects model will be selected to generate the MD or SMD with its corresponding 95% CI.

### 2.9 Assessment of publication bias

If more than 10 original studies are included, funnel plots will be made according to the data of the included studies to observe publication bias. If the funnel plot is asymmetric, it indicates publication bias. We will discuss the sources and explanations of bias.

### 2.10 Data synthesis

A forest plot for each parameter will be constructed to illustrate the weight ratio of each incorporated study. All statistical analyses will be carried out using the RevMan5.3 software, and the significance threshold will be a 2-sided *P* <.05.

### 2.11 Sensitivity analysis

In order to evaluate the sensitivity of the meta-analysis, studies will be excluded one by one, and the differences of the combing effects before and after exclusion will be compared, and if the pooled outcomes are found to have been reversed after the exclusions, the outcomes may be unstable.

### 2.12 Subgroup analysis

When heterogeneity is high, if the necessary data are available, subgroup analyses will be conducted for different comparators separately. In addition, if the expected efficacy is not observed in all the subjects, subgroup analysis could help us show whether the treatment is effective in some specific subgroups. At the same time, subgroup analysis can also help us to show whether the therapeutic effect is better in particular subjects if it is found to be effective in all subjects.

### 2.13 Grading the quality of evidence

The Grading of Recommendations Assessment, Development, and Evaluation (GRADE) method is used to evaluate the quality of evidence for each outcome of meta-analysis. The GRADE Working Group recommended that the quality of evidence can be classified into 4 levels: high (++++), moderate (+++), low (++), and very low (+). Evidence quality is generally judged on the basis of risk of bias, inconsistency, indirectness, inaccuracy and publication bias. We can evaluate it on this page: https://gradepro.org/.

## 3 Discussion

The protocol of this systematic review and meta-analysis study aims to assess the efficacy, safety and cost benefits of small splint in the treatment of distal radius fractures in children. In the meantime, we have tried our best to search and found that this study is the first systematic review and meta-analysis concerning this topic, integrating the latest and most comprehensive clinical evidence in this field, hoping to inspire more peer experts and doctors to carry out as many relevant studies as possible in the future. Moreover, some evidences can be obtained to further guide the selection and suitable interventions by analyzing and integrating the existing clinical studies.

In addition, the systematic review and meta-analysis of GRADE evidence grading assessment are more conducive to clinical decision-making and guideline transformation. Meanwhile, this study has been registered on PROSPERO, which makes it more transparent and trustworthy.

## Acknowledgment

This study is supported by the project—“Seedling Fund Cultivation” funded by China Academy of Chinese Medical Sciences(ZZ11–084).

## Author contributions

**Conceptualization:** Xin Cui, Long Liang.

**Data curation:** Jiani Liu, Zhibo Wang, Yue Zhang.

**Methodology:** Yanming Xie, Xu Wei, Xing Liao.

**Resources:** Yongzhong Cheng, Yachao Du, Hongyan Zhang.

**Software:** Shiheng Wang, Guangwei Liu, Yaliang Tian.

**Supervision:** Yongyao Li, Hao Cheng.

**Writing – original draft:** Xin Cui, Long Liang, Xu Wei, Xing Liao.

**Writing – review & editing:** Xin Cui, Long Liang, Yanming Xie, Yongyao Li, Hao Cheng.
